# Sudden gains in modular CBT for mental health disorders in children and young people with epilepsy

**DOI:** 10.1111/jcpp.14164

**Published:** 2025-03-20

**Authors:** Alvin Richards‐Belle, Daniela Linton, J. Helen Cross, Isobel Heyman, Emma Dalrymple, Bruce Chorpita, Sophia Varadkar, Mariam Shah, MICE Study Group, Sarah Byford, Anna Coughtrey, Peter Fonagy, Tamsin Ford, Rona Moss‐Morris, Colin Reilly, Jonathan A. Smith, Terence Stephenson, Roz Shafran, Sophie Bennett

**Affiliations:** ^1^ Division of Psychiatry University College London London UK; ^2^ UCL Great Ormond Street Institute of Child Health University College London London UK; ^3^ Great Ormond Street Hospital for Children NHS Foundation Trust London UK; ^4^ Cambridge and Peterborough NHS Foundation Trust Cambridge UK; ^5^ Department of Psychology University of California Los Angeles CA USA; ^6^ Institute of Psychiatry, Psychology and Neuroscience King's College London London UK

**Keywords:** Children and young people, epilepsy, long‐term conditions, mental health disorders, CBT

## Abstract

**Background:**

Sudden gains (rapid, large, stable improvements in symptoms) are common in psychological therapy and are associated with favourable outcomes, but no studies have investigated sudden gains in children and young people (CYP) with a chronic physical condition.

**Methods:**

Within‐group study nested in the Mental Health Intervention for Children with Epilepsy (MICE) randomised trial of modular cognitive‐behavioural therapy for CYP with epilepsy, utilising goal‐based outcomes (GBOs) and standardised session‐by‐session measures (including the brief parental self‐efficacy scale and Strengths and Difficulties Questionnaire [SDQ] session‐by‐session measure). The occurrence and potential predictors of sudden gains, and the association of sudden gains with outcomes at final session and follow‐up were investigated using multivariable logistic and linear regression.

**Results:**

Among 147 participants (mean age: 10.4 years, 49% female) and across nine measures, 39% experienced between two and four sudden gains, most frequently on the mean GBO (occurrence, 44.9%). Characteristics such as intellectual disability, pretreatment scores and the number of sessions received were associated with significantly greater odds of sudden gains in some measures, whereas nonwhite ethnicity and nonemployment of the primary caregiver were associated with reduced odds. Sudden gains were associated with favourable final‐session scores for mean GBO (GBO, adjusted mean difference [aMD]: 0.9, 95% CI: 0.3 to 1.6, *p* = .004, *D* = 0.63), parental self‐efficacy (aMD: 1.2, 95% CI, 0.1 to 2.4, *p* = .027, *D* = 0.37) and the SDQ session‐by‐session measure (aMD: ‐1.7, 95% CI, −3.0 to −0.3, *p =* .014, *D* = ‐0.44), but not with 6‐month adjusted SDQ total difficulties scores.

**Conclusions:**

Sudden gains were common in this population, occurring most frequently on personalised measures, and were associated with favourable final‐session scores. Personalised measures taken at each session with a focus on sudden gains may be a useful adjunct to treatment. Future research and clinical practice should investigate how to increase the occurrence of sudden gains in CYP with long‐term conditions receiving psychological therapy.

## Introduction

Improving the mental health of Children and Young People (CYP) with long‐term physical health conditions (LTCs) is a national priority in the United Kingdom (NHS, 2019). Epilepsy, the most common significant long‐term neurological condition in CYP (Aaberg et al., 2017), is associated with very high levels of psychological morbidity (Moore et al., 2019) which often is unmet in current service provision (Mahendran, Speechley, & Widjaja, 2017). Studying predictors of outcomes from psychological therapies delivered to CYP with LTCs, such as epilepsy, could inform future intervention development and optimisation, expanding the evidence base for addressing psychological morbidity in this population.

One way to expand this evidence base is by studying processes of change during psychological therapy and their relation to outcomes. Research consistently shows that rapid, large, stable improvements in symptoms between sessions (which are usually weekly), or ‘*sudden gains*’, are common during therapy, with approximately one third (34.7%) of individuals experiencing sudden gains (Shalom & Aderka, 2020). This concept was introduced by Tang and DeRubeis (1999), who proposed three quantitative criteria for identifying sudden gains – a change in symptom score must be large: (a) in absolute terms, (b) relative to previous symptom score and (c) relative to symptom fluctuation. Tang and DeRubeis (1999) proposed that cognitive changes in a critical therapy session might trigger a sudden gain – leading to an ‘upward spiral’ of improved therapeutic alliance and further cognitive changes, and ultimately, recovery.

Despite ongoing debate on how best to operationalise the criteria (see Appendix S1: page 2 for details), meta‐analyses show that individuals who experience a sudden gain in therapy are more likely to achieve a favourable outcome posttherapy and later follow‐ups (over and above the effects of treatment modalities, settings and disorders) compared to those without a sudden gain (Aderka, Nickerson, Bøe, & Hofmann, 2012; Shalom & Aderka, 2020). However, to date, most research has focused on adults; in the most recent systematic review, only eight of 57 treatment conditions were in CYP (Shalom & Aderka, 2020). Moreover, to the best of our knowledge, a study of 26 adults with cancer and depression (Hopko, Robertson, & Carvalho, 2009) is the only study of sudden gains in people (of any age) with an LTC, providing initial evidence that sudden gains may be applicable to populations with physical health comorbidity, but further research is needed with much more diverse samples of people with LTCs, including CYP.

Eight studies have investigated sudden gains in CYP across various diagnoses and treatment modalities (Aderka, Appelbaum‐Namdar, Shafran, & Gilboa‐Schechtman, 2011; Dour, Chorpita, Lee, & Weisz, 2013; Durland, Wyszynski, & Chu, 2018; Gaynor et al., 2003; Gibby, 2015; Mechler et al., 2021; Mychailyszyn, Carper, & Gibby, 2018; Storch et al., 2019). The largest study, involving 161 participants with diverse mental disorders, compared Modular Approach to Therapy for Children with Anxiety, Depression, Trauma and Conduct Problems (MATCH‐ADTC) (Chorpita & Weisz, 2009), standard manual treatment and usual care (Dour et al., 2013). The authors reported that sudden gains occurred more frequently when assessed using an idiographic (i.e. personalised, individual) measure, as compared to a standardised measure, and recommended further evaluation of idiographic measures (Dour et al., 2013). Overall, these studies provide inconsistent evidence on whether CYP characteristics are associated with the occurrence of sudden gains. Whilst some reported that sudden gains were associated with favourable outcomes posttherapy and/or at follow‐up (Aderka et al., 2011; Dour et al., 2013; Gaynor et al., 2003; Storch et al., 2019), others did not (Durland, Wyszynski, & Chu, 2018; Gibby, 2015; Mechler et al., 2021). As sudden gains have previously been associated with therapeutic outcome, they may provide insight into mechanisms of action. For example, if they occur early in therapy – as has generally been observed in other CYP studies of sudden gains (Aderka, Appelbaum‐Namdar, Shafran, & Gilboa‐Schechtman, 2011; Dour et al., 2013; Durland, Wyszynski, & Chu, 2018; Gaynor et al., 2003; Gibby, 2015; Mechler et al., 2021; Mychailyszyn, Carper, & Gibby, 2018; Storch et al., 2019) – then this may suggest that an aspect of the therapy earlier in treatment, such as psychoeducation, was a key ingredient in facilitating change. Knowing who is more likely to experience sudden gains may also help to further modify treatments for CYP with different demographic or clinical characteristics (i.e. how can we increase the likelihood of sudden gains).

A recent randomised clinical trial (RCT) found that a modular Mental health Intervention for CYP with Epilepsy (MICE), based on the MATCH‐ADTC protocol (Chorpita & Weisz, 2009), was superior to the control arm on clinical outcomes (Bennett et al., 2024). As part of the trial, participants completed session‐by‐session measures for each week of therapy, in addition to measures at baseline, 6‐ and 12‐months postrandomisation. There is therefore the potential to investigate the applicability of sudden gains criteria for CYP with LTCs within this trial. This is the first full‐scale RCT to directly address mental health disorders in children and young people with epilepsy, and therefore provides significant scope to investigate mechanisms of action to further improve treatments and associated outcomes. This is extremely important given the high rates of mental health difficulties within this group together with high levels of unmet need (Mahendran et al., 2017; Moore et al., 2019). In addition, the most common mental health difficulties in CYP with epilepsy are the same as those in children without epilepsy (anxiety, depression and behavioural difficulties) often cooccurring with neurodivergence (Davies, Heyman, & Goodman, 2003); and therefore, insights into mechanisms of action in CYP with epilepsy may be similar to those for CYP with other LTCs and without LTCs. The population within the MICE trial included children and young people aged 3–18 years who had intellectual disability, were neurodivergent and presented with different mental health difficulties (anxiety, depression and behavioural difficulties). There is therefore scope to investigate moderators of change that have not been possible to investigate in other CYP studies of sudden gains.

This study sought to address three main questions:

1. *Occurrence of sudden gains*: How many CYP with mental health disorders in the context of epilepsy experience sudden gains during a modular psychological intervention as assessed by both idiographic and standardised measures?
2. *Characteristics associated with sudden gains*: Are there CYP characteristics associated with the occurrence of sudden gains?
3. *Sudden gains and outcome*: Do participants who experience a sudden gain have a more favourable outcome at the end of therapy and at 6‐month follow‐up?

## Methods

### Design

This was a within‐group study nested in the multicentre MICE RCT (ISRCTN57823197). MICE received ethical approval from South Central – Oxford A Research Ethics Committee (reference: 18/SC/0250). All participants, or their parents/legal guardians, provided written informed consent.

### Participants

Eligibility criteria included: Attending an NHS paediatric epilepsy clinic; Aged 3–18 years; Strengths and Difficulties Questionnaire (SDQ) total difficulties score ≥14 and impact score ≥2; Meeting Diagnostic and Statistical Manual of Mental Disorders, Fifth Edition (DSM‐5) criteria for a mental health disorder (e.g. depression, anxiety, disruptive behaviour, or trauma and stress‐related disorders) identified by a clinically rated Development and Well‐Being Assessment (DAWBA) (R. Goodman, Ford, Richards, Gatward, & Meltzer, 2000); and having a parent/carer willing to participate (Bennett, Cross, et al., 2021).

This sub‐study used data from participants randomised to MICE therapy. Therapy consisted of up to 22 sessions (including two booster sessions) of cognitive‐behavioural therapy and/or behavioural parenting therapy, delivered via telephone/video call by nonmental health professionals (with supervision from qualified clinical psychologists) over a 6‐month period, in addition to usual care. Therapy targeted both internalising and externalising symptoms, with flexible use of modules, allowing therapists to focus on multiple presenting problems, tailored to participant needs. Details of therapists, their training, supervision and competence, and adherence are available elsewhere (Bennett, Au, et al., 2021; Bennett et al., 2024; Coughtrey et al., 2024; Shafran et al., 2020). Therapy was delivered to CYP and/or parents, according to age and capacity.

### Measures

Sudden gains were investigated in session‐by‐session measures, which families (parents and/or CYP, according to age and capacity) were asked to complete before the start of each session and email to the therapist. The 6‐month SDQ total difficulties score is the MICE trial primary outcome; we investigated whether sudden gains were associated with this standardised outcome.

#### Goal‐based outcomes (GBOs)

Up to three idiographic GBOs were codeveloped at the start of therapy between the participant and therapist, with progress towards each goal rated on a scale of 1 (low progress) to 10 (high progress) in subsequent sessions (Edbrooke‐Childs, Jacob, Law, Deighton, & Wolpert, 2015). All goals in the behavioural treatment were developed between the parent and therapist. Given the wide age range of participants and high levels of intellectual disability, parents were involved in setting the goals for other participants where appropriate. An example of a goal for disruptive behaviour might be ‘to react calmy to situations out of their control, e.g. leaving an activity they like, waiting their turn, or can't find something they are looking for’. Examples for anxiety might include ‘to ask my teacher for help every time I am stuck in class’ or, for the parent, ‘for X's fear and anxiety around bedtime to reduce ‐ able to settle in bed within 40 minutes and go to sleep without me coming back in the room’. An example for low mood might include ‘to go back to playing football in my club’. GBOs have acceptable internal consistency (Edbrooke‐Childs et al., 2015).

#### Oppositional defiant disorder‐parent reported measure (ODD‐p)

Disruptive behaviour was assessed using the eight‐item ODD‐p measure of behavioural difficulties. Items, based on DSM‐IV criteria, were rated 0 (not true), 1 (somewhat true) or 2 (certainly true) and summed for an overall score, with higher scores indicative of greater disruptive behaviour (Child Outcomes Research Consortium, 2023). To the best of our knowledge, there are no validation studies of the ODD‐p.

#### Revised Child Anxiety and Depression Scale (RCADS)

Generalised anxiety, separation anxiety, social anxiety, panic and low mood/depression were all assessed using RCADS (Chorpita, Moffitt, & Gray, 2005; Chorpita, Yim, Moffitt, Umemoto, & Francis, 2000) subscales, of which reliabilities range from acceptable to good (Piqueras, Martín‐Vivar, Sandin, San Luis, & Pineda, 2017). Items were rated 0 (never), 1 (sometimes), 2 (often) or 3 (always) and summed for an overall score, with higher scores indicative of greater symptoms.

#### Seizure impact

The single investigator‐derived item ‘How much of an impact have my child's seizures had on my child's life?’ was rated on a scale from 1 (no impact) to 10 (significant negative impact) for those with active seizures. Among participants randomised to receive MICE therapy in the trial, 63% reported having seizures in the past 3 months (Bennett et al., 2024).

#### Brief parental self‐efficacy scale (BPSES)

Parental self‐efficacy was assessed using the five‐item BPSES – a standardised measure describing a parent's belief in their ability to perform the parenting role successfully and which comprises three domains (knowledge, ability and outcome). Items were rated on a scale from 1 (strongly disagree) to 5 (strongly agree) and summed for an overall score. Higher scores indicate greater self‐efficacy. BPSES has satisfactory internal consistency (Woolgar, Humayun, Scott, & Dadds, 2023).

#### SDQ session‐by‐session (S×S)

SDQ S×S scale assesses the impact that emotional and behavioural symptoms have on home life, friendships, ability to learn/work and leisure activities, with additional questions on symptom improvement and hope for the future (Goodman, 1997). Items were rated 0 (not at all), 1 (only a little), 2 (a medium amount), 3 (a great deal) and summed for an overall score, with higher scores suggestive of greater levels of distress/impairment.

Goal‐based outcomes (GBOs), seizure impact, parental self‐efficacy and SDQ S×S were intended to be assessed in all participants at every session; ODD‐p and RCADS subscales were administered if and when relevant to the participant's therapy (see Table S1 for further detail on session‐by‐session measures).

#### SDQ

The SDQ is a widely used and validated brief behavioural screening questionnaire, comprising 25 items across five subscales (emotional, conduct problems, hyperactivity/inattention, peer relationship problems and prosocial behaviour), with the first four scales summed to provide a total difficulties score, ranging from 0 to 40. The SDQ has good internal consistency (mean Cronbach alpha 0.73). Scores above the 90th percentile are predictive of a substantially raised probability of independently diagnosed psychiatric disorders (Goodman, 2001). The SDQ was administered to parents (Goodman, 1997) at six months postrandomisation by an independent member of the research team blinded to treatment condition, with electronic or telephone completion.

### Statistical analysis

All statistical tests were two‐sided, used a 5% significance level and were reported with 95% confidence intervals (CIs). Analyses were conducted using R version 4.3.1 (R Core Team, 2024). The analysis population was all participants randomised to MICE therapy who had not withdrawn consent. Participants were excluded if they received fewer than six sessions (a trial deviation).

Baseline patient, family and therapy characteristics are presented in a table and include age, sex, ethnicity, quintile of deprivation, comorbidities, number of siblings, employment and marital status of the primary caregiver; total number of sessions received, primary mental health disorder, and whom therapy and measures were completed with.

Session‐by‐session measures were scored according to instructions (see Table S1). Using the *suddengains* R package (Wiedemann, Thew, Stott, & Ehlers, 2020), we constructed an operational definition of sudden gains, based on the three criteria used in Tang and DeRubeis (1999), as follows. We calculated and used the reliable change index (RCI) (Jacobson & Truax, 1991) for measures (Table S1) to define large absolute change (criterion one) on each measure and conservatively rounded up to the next whole number, given that scales used whole numbers (Storch et al., 2019). Criterion two was applied as per Tang and DeRubeis (1999) (i.e. the gain must represent at least 25% of the pregain score). For criterion three (stability), a two‐sample *t*‐test was used to determine if the mean scores of the two/three sessions after the potential gain were significantly different from the two/three before. Accounting for potential missing data and enabling maximal use of data points, significance was determined against a critical *t* value of either >2.776 (when three measurements were available both before and after the potential gain), >3.182 (when one measurement was missing before or after the potential gain) or >4.303 (where one measurement was missing both before and after the potential gain) (Lutz et al., 2013). Sudden gains were investigated on session‐by‐session measures completed by >20 participants. Missing session‐by‐session data were not imputed as imputation could lead to additional sudden gains that were not reported by participants (Mechler et al., 2021). We report the occurrence rate, modal session number during which a sudden gain first occurred, magnitude of gains and proportions experiencing multiple and reversal of sudden gains [defined as losing 50% or more of the sudden gain at any point after experiencing a sudden gain (Tang & DeRubeis, 1999)]. Sudden losses, the inverse of the sudden gains criteria (i.e. a sudden worsening in scores), were also examined in a similar way to the reporting of sudden gains.

Multivariable logistic regression was used to estimate the association of participant characteristics with the occurrence of sudden gains across measures, with adjusted odds ratios (aOR) reported. Linear regression was used to estimate the associations of sudden gains with the final session‐by‐session score (i.e. on the measure in which the sudden gain was identified) and the 6‐month SDQ total difficulties score, with unadjusted and adjusted mean differences (aMD) reported. All models were adjusted for sex, age group, presence of autism spectrum disorder or intellectual disability, ethnicity (white vs. other), primary caregiver employment status, primary mental health disorder (disruptive behaviour vs. anxiety/depression), pretreatment score (i.e. on the relevant measure) and total number of therapy sessions received. Models investigating the 6‐month outcome were also adjusted for the baseline SDQ total difficulties score. We calculated Cohen's D effect sizes for interpretability.

## Results

### Sample characteristics

**Figure 1:**
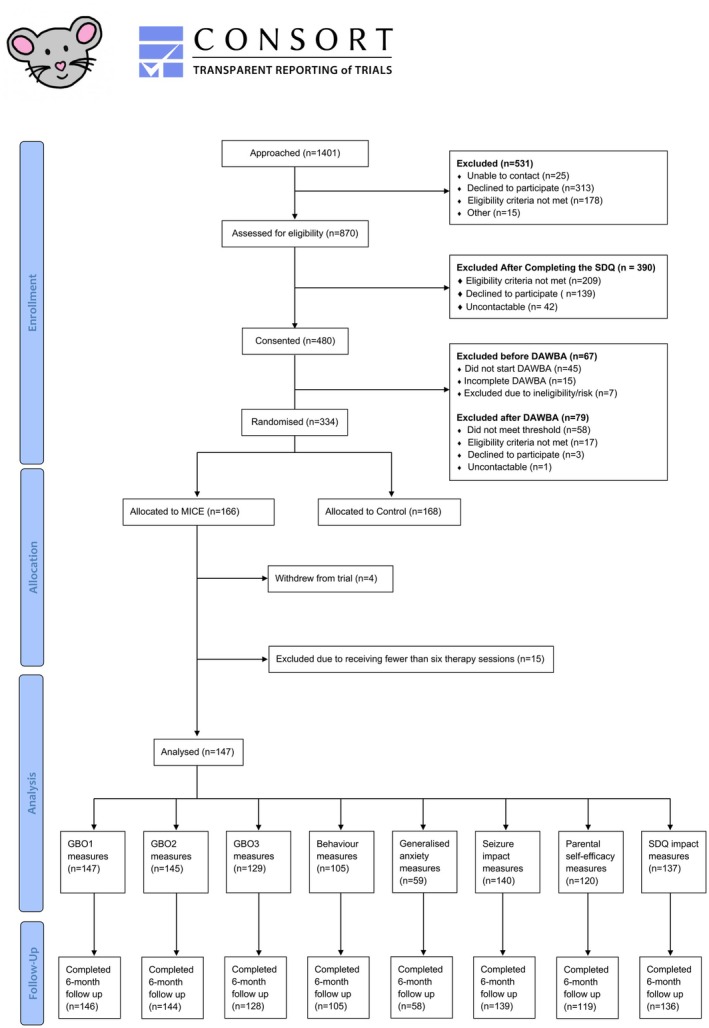
Flow chart/CONSORT diagram

In total, 334 participants were recruited into the MICE trial between August 2019 and February 2022; 166 were randomised to receive MICE therapy, four withdrew consent, and 15 received fewer than six sessions, resulting in 147 included in this study (Figure 1). The sample had a mean (*SD*) age of 10.4 (3.5) years, almost half (49.0%) were female, the majority white (74.8%), around a quarter (23.8%) had autism spectrum disorder and around two‐fifths (41.5%) had intellectual disability (Table 1). Participants received a median (IQR) of 19 (15–21) sessions (including booster sessions). Disruptive behaviour was the most frequent primary mental health disorder (57.8%), followed by anxiety (38.1%). For most participants (>93%), a parent was involved in therapy and in the completion of session‐by‐session measures. Depression, social anxiety and panic session‐by‐session measures had <20 responses and were not analysed further (Table S2).

**Table 1:** Sample characteristics

Characteristics	*N* = 147
Demographics	
Age at randomisation	
Median (IQR)	10.0 (8.0, 13.0)
Mean (*SD*)	10.4 (3.5)
Age at randomisation – category,^a^ *n* (%)	
<11 years	79 (53.7%)
≥11 years	68 (46.3%)
Age at epilepsy diagnosis, median (IQR)	5.0 (2.0, 8.0)
Sex, *n* (%)	
Female	72 (49.0%)
Male	75 (51.0%)
Ethnicity, *n* (%)	
Asian	9 (6.1%)
Black	4 (2.7%)
Mixed	17 (11.6%)
Other	4 (2.7%)
White	110 (74.8%)
Prefer not to say	3 (2.0%)
Comorbidities, *n* (%)	
Autism spectrum disorder	36 (24.5%)
Intellectual disability	61 (41.5%)
Index of multiple deprivation (quintile), *n* (%)	
1 – most deprived	13 (9.4%)
2	39 (28.3%)
3	31 (22.5%)
4	18 (13.0%)
5 – least deprived	37 (26.8%)
Unknown	9
Family	
Number of siblings in household, *n* (%)	
0	24 (16.3%)
1	65 (44.2%)
2	38 (25.9%)
3+	20 (13.6%)
Employment status of primary caregiver, *n* (%)	
Employed	91 (61.9%)
Not employed	56 (38.1%)
Marital status of primary caregiver, *n* (%)	
Divorced/Separated	7 (4.8%)
Living with Partner	17 (11.6%)
Married	97 (66.0%)
Single	22 (15.0%)
Other/Prefer not to say	4 (2.7%)
Trial/intervention	
Total number of sessions received, median (IQR)	19 (15, 21)
Primary problem area, *n* (%)	
Anxiety	56 (38.1%)
Depression	6 (4.1%)
Disruptive Behaviour	85 (57.8%)
Therapy completed with, *n* (%)	
CYP	9 (6.2%)
Parent	107 (73.3%)
Parent and CYP	30 (20.5%)
Unknown	1
Session‐by‐session measures completed by, *n* (%)	
CYP	8 (5.5%)
Parent	123 (84.2%)
Parent and CYP	15 (10.3%)
Unknown	1

CYP, child or young person.

^a^ Categories as per Bennett et al. (2024).

### Occurrence of sudden gains

Data from 2,601 sessions were analysed; sudden gains and losses are summarised (including the occurrence rate, magnitude of gain/losses and rate of reversals) in Tables 2 and S3, respectively.

**Table 2:** Sudden gains

Domain	*N* ^a^	Session intervals analysed, *n*	Total gains, *n*	Occurrence rate, *n* (%)	Session no, mode	Multiple, *n* (%)	Magnitude, mean (*SD*)	Reversal, *n* (%)
GBO mean	147	1,898	80	66 (44.90)	2	14 (9.52)	2.74 (0.91)	15 (22.73)
Disruptive behaviour	105	1,065	41	33 (31.43)	5	8 (7.62)	4.06 (2.66)	19 (57.58)
Generalised anxiety	59	605	17	16 (27.12)	8	1 (1.69)	3.56 (1.21)	5 (31.25)
Separation anxiety	27	247	6	5 (18.52)	6	1 (3.70)	4.00 (2.35)	3 (60.00)
Seizure impact	140	1,427	30	26 (18.57)	2	4 (2.86)	3.62 (1.70)	14 (53.85)
Parental self‐efficacy	120	1,258	45	41 (34.17)	4	4 (3.33)	3.34 (1.48)	14 (34.15)
SDQ S×S	137	1,292	45	35 (25.55)	10	8 (5.84)	4.03 (2.44)	18 (51.43)

GBO, goal‐based outcome; SDQ, strengths and difficulties questionnaire.

^a^ Participants are included in the *n* for a specific measure if they received at least six sessions, had not withdrawn consent and had >1 response on the relevant scale through the course of therapy.

Almost half of the participants (44.9%) experienced a sudden gain in their mean GBO score, with around a quarter of later experiencing a reversal of their gain (22.7%). Less than 5% experienced a sudden loss in the mean GBO, but of those that did, most subsequently experienced a reversal of the loss (85.7%) (Table S3). For participants completing the behaviour measure, 31.4% experienced a sudden gain on the disruptive behaviour measure (i.e. representing improvements in symptoms). For participants completing the generalised anxiety measure, 27.1% experienced a sudden gain on the generalised anxiety measure. The rate of reversal was higher on the behaviour measure than on the generalised anxiety measure (57.6% vs. 31.3%). The lowest sudden gain occurrence rates were in seizure impact (i.e. improvements in seizure impact) (18.6%) and separation anxiety (18.5%).

**Figure 2:**
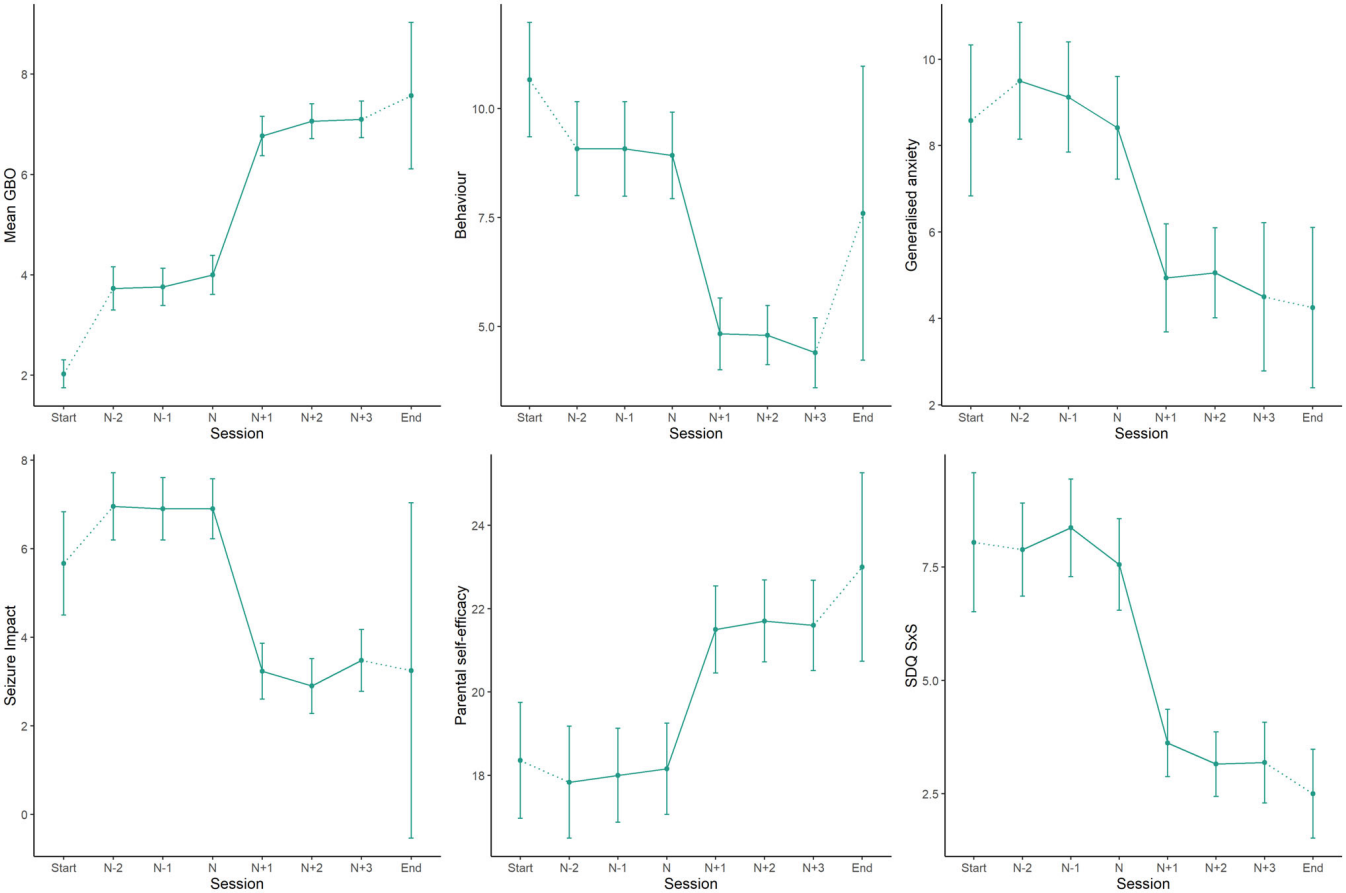
Sudden gains across mean goal‐based outcome, disruptive behaviour, generalised anxiety, seizure impact, brief parental self‐efficacy and SDQ S×S score.* (* N represents the pregain session, and *N* + 1 represents the session in which the sudden gain occurred. For GBO mean and parental self‐efficacy, a higher score indicates a better outcome. For disruptive behaviour, generalised anxiety, seizure impact and SDQ S×S, a lower score indicates a better outcome)

Most initial mean GBO, parental self‐efficacy and seizure impact sudden gains occurred very early in therapy (modal session number, range 2–4), whereas gains in other standardised measures occurred most frequently between sessions 5 and 10 (gains in SDQ S×S occurred at the latest point). Across measures, 39% experienced between two and four sudden gains (Table S4). The trajectory of sudden gains across six measures is shown in Figure 2.

Sudden losses were most frequent on the generalised anxiety, disruptive behaviour and seizure impact measures. Very few participants experienced multiple sudden losses on a given measure, and the rate of reversal was generally much higher when compared with sudden gains. Compared to sudden gains, sudden losses most frequently occurred later in therapy (modal session number, range: 8–13), apart from generalised anxiety, which occurred most frequently in session 2. Across measures, those experiencing a sudden loss only typically experienced one (Table S5).

### Associations of characteristics with sudden gains

**Figure 3:**
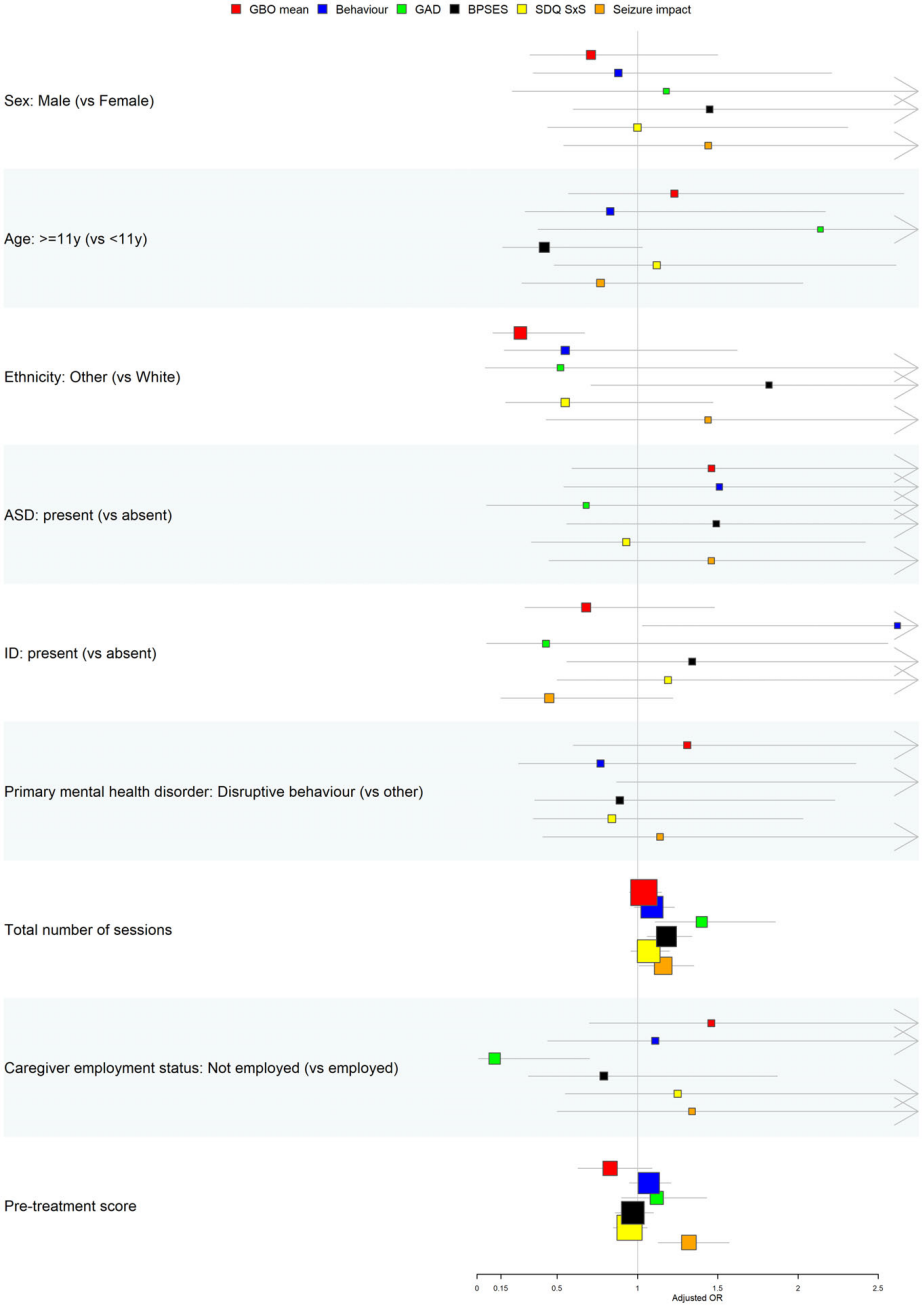
Adjusted associations between characteristics and occurrence of sudden gains across measures. ASD, autism spectrum disorder; ID, intellectual disability. Forest plot of the adjusted odds ratio for experiencing a sudden gain across each of the domains (see legend) for each characteristic relative to its reference (shown in brackets). Point estimates are indicated by squares, with lines on either side indicating the confidence interval. Estimates to the right of the no effect line (vertical line at 1) indicate greater odds of a sudden gain, while estimates to the left indicate reduced odds. If the confidence interval crosses 1, then the association is not statistically significant at the 0.05 level. All models are adjusted for: sex, age group, ethnicity, presence of autism spectrum disorder, presence of intellectual disability, total number of sessions, primary mental health disorder (disruptive behaviour vs. anxiety/depression), employment status of primary caregiver and the relevant pretreatment score

Multivariable logistic regression indicated that, compared to white ethnicity, other ethnicities were associated with a significantly reduced odds of experiencing a sudden gain in the mean GBO (aOR, 0.27, 95% CI, 0.10–0.67, *p* = .007) (Figure 3, Table S6). Intellectual disability, compared to no intellectual disability, was significantly associated with greater odds of experiencing a sudden gain on the disruptive behaviour measure (aOR, 2.62, 95% CI, 1.03–7.01, *p* = .046). Nonemployment, compared to employment, of the primary caregiver was significantly associated with reduced odds of experiencing a sudden gain in the generalised anxiety measure (aOR, 0.11, 95% CI, 0.01–0.70, *p* = .039). The pretreatment seizure impact score was significantly associated with the occurrence of sudden gains on the seizure impact measure. Specifically, a one‐unit increase in seizure impact score at baseline was associated with greater odds of a sudden gain (i.e. improvement in seizure impact) (aOR, 1.32, 95% CI, 1.13–1.57, *p* = .001). The total number of sessions that a participant received was a significant predictor of sudden gains, with each additional session associated with increased odds of a sudden gain in generalised anxiety (aOR, 1.40, 95% CI, 1.11–1.86, *p* = .010), seizure impact (aOR, 1.16, 95% CI, 1.01–1.35, *p* = .041) and parental self‐efficacy (aOR, 1.18, 95% CI, 1.06–1.34, *p* = .004).

### Associations of sudden gains with final‐session and 6‐month outcome scores

Experiencing a sudden gain in the mean GBO was significantly associated with a more favourable final‐session mean GBO score, compared to those without a sudden gain (aMD: 0.9, 95% CI: 0.3 to 1.6, *p* = .004, *D* = 0.63) (Table 3). Similarly, a sudden gain in SDQ S×S was associated with favourable final‐session SDQ S×S scores (aMD: −1.7, 95% CI, −3.0 to −0.3, *p =* .014, *D* = −0.44). Sudden gains in parental self‐efficacy were associated with higher final‐session parental self‐efficacy scores in adjusted analysis (aMD: 1.2, 95% CI, 0.1 to 2.4, *p* = .027, *D* = 0.37). Experiencing a sudden gain in any of the measures was not significantly associated with SDQ total difficulties scores at 6 months, having adjusted for baseline SDQ scores (Table 3).

**Table 3:** Unadjusted and adjusted associations between sudden gains and final‐session scores and 6‐month outcomes across measures^a^

Domain	Final‐session score^b^					6‐month SDQ total difficulties score^c^				
	No sudden gain	Sudden gain	Effect of sudden gains			No sudden gain	Sudden gain	Effect of sudden gains		
	Mean (*SD*) [*n*]	Mean (*SD*) [*n*]	Unadjusted mean difference	Adjusted mean difference^d^	Adjusted Cohen's *D* ^d^	Mean (*SD*) [*n*]	Mean (*SD*) [*n*]	Unadjusted mean difference	Adjusted mean difference^d^	Adjusted Cohen's *D* ^d^
GBO mean	7.4 (2.2) [81]	8.3 (1.4) [66]	1.0 (0.3, 1.6) [0.002]	0.9 (0.3, 1.6) [0.004]	−0.52	17.5 (6.3) [81]	16.8 (6.2) [65]	−0.7 (−2.8, 1.4) [0.50]	−0.5 (−2.7, 1.7) [0.65]	0.11
Behaviour	6.0 (3.9) [72]	5.4 (3.7) [33]	−0.7 (−2.3, 0.9) [0.41]	−1.0 (−2.6, 0.5) [0.17]	0.17	17.9 (6.0) [72]	16.8 (6.6) [33]	−1.1 (−3.6, 1.5) [0.41]	−1.8 (−4.4, 0.7) [0.16]	0.17
Generalised anxiety	5.2 (3.5) [43]	5.2 (2.8) [16]	0.1 (−1.9, 2.0) [0.95]	−0.0 (−2.2, 2.2) [>0.99]	−0.02	16.3 (5.5) [42]	18.6 (5.8) [16]	2.2 (−1.0, 5.5) [0.17]	3.1 (−0.8, 7.0) [0.11]	−0.40
Seizure impact	3.2 (2.7) [114]	3.0 (2.4) [26]	−0.1 (−1.3, 1.0) [0.79]	−0.8 (−1.9, 0.3) [0.15]	0.06	17.4 (6.3) [113]	15.5 (5.1) [26]	−1.9 (−4.5, 0.7) [0.15]	−2.6 (−5.4, 0.2) [0.064]	0.31
Parental self‐efficacy	21.3 (3.3) [79]	22.4 (2.9) [41]	1.1 (−0.1, 2.3) [0.072]	1.2 (0.1, 2.4) [0.027]	−0.35	16.5 (5.9) [78]	18.5 (6.5) [41]	2.0 (−0.3, 4.4) [0.085]	1.7 (−0.8, 4.2) [0.17]	−0.33
SDQ S×S	5.8 (3.8) [102]	3.7 (3.9) [35]	−2.1 (−3.6, −0.7) [0.005]	−1.7 (−3.0, −0.3) [0.014]	0.56	17.4 (6.1) [101]	15.5 (6.4) [35]	−1.9 (−4.3, 0.5) [0.13]	−1.4 (−3.6, 0.9) [0.23]	0.30

GBO, goal‐based outcome; SDQ, strengths and difficulties questionnaire.

^a^ Separation anxiety was not analysed given the small number of sudden gains identified on that measure (*n* = 5).

^b^ For mean GBO and parental self‐efficacy, a higher score indicates a better outcome. For disruptive behaviour, generalised anxiety, seizure impact and SDQ S×S; a lower score indicates a better outcome.

^c^ Strengths and difficulties questionnaire (SDQ) total difficulties scores are reported for participants that responded to the 6‐month follow‐up. SDQ total difficulties scores range 0–40; higher scores indicate a greater probability of a diagnosable mental health disorder.

^d^ Adjusted for: sex, age group, ethnicity, presence of autism spectrum disorder, presence of intellectual disability, total number of sessions, primary mental health disorder (disruptive behaviour vs. anxiety/depression), employment status of primary caregiver and the relevant pretreatment score. Models investigating the 6‐month outcome were also adjusted for the baseline SDQ total difficulties score.

## Discussion

This study significantly extends the sudden gains literature by providing the first investigation of sudden gains in psychological therapy for CYP with mental health disorders in the context of an LTC (epilepsy). We showed that sudden gains occurred frequently across a range of measures during a remotely delivered, evidence‐based, modular psychological intervention delivered by nonmental health professionals to CYP attending epilepsy services in an RCT – with many experiencing multiple sudden gains across measures.

Sudden gains occurred more frequently on idiographic measures, compared to standardised measures. This closely replicates the finding of Dour et al. (2013) in which, overall, 33% experienced a sudden gain in average Top Problems Assessment scores – but which increased to 47% when considering the modular treatment group only (also based on the MATCH‐ADTC protocol) and is very similar to the 45% that we reported on a comparable idiographic measure, the mean GBO. These results allude to the idea that standardised measures may not tap into the issues of most importance to CYP and their families during therapy and demonstrate the importance of capturing both idiographic and standardised measures in practice (Jacob, Edbrooke‐Childs, Flannery, Segal, & Law, 2022). We did not review the specific content of goals; detailed studies assessing whether certain types of GBOs (O'Reilly, McKenna, & Fitzgerald, 2022) are more or less associated with sudden gains (and outcomes) are warranted. Sudden losses were less common and, reassuringly, the majority of sudden losses were reversed during the course of therapy.

We found evidence for a small number of characteristics being associated with sudden gains in some measures. Compared to white ethnicity, participants of other ethnicities were significantly less likely to experience sudden gains in the mean GBO – a finding consistent with Storch et al. (2019) and Gibby (2015), despite differences in disorders, settings and measures – suggesting this finding is not unique to the LTC population or the modular therapy used in this study. Whilst our sample had more ethnic diversity than Storch et al. (2019), we were not able to disaggregate other ethnicities due to low absolute numbers. Meta‐analyses suggest that ethnicity does not impact treatment outcomes (Cougle & Grubaugh, 2022), though diverse groups have been underrepresented in cognitive‐behavioural therapy trials (Horrell, 2008). Further research is needed to evaluate the applicability of sudden gains and other processes of change in diverse populations. The total number of sessions was the only characteristic that was a significant predictor of sudden gains across multiple domains, which intuitively makes sense given that more sessions provide greater opportunities for sudden gains to occur (although this was not found by Durland, Wyszynski, & Chu, (2018)). However, like other studies, most characteristics assessed, including age and primary disorder, were typically not consistently associated with sudden gains, especially across measures. Other authors have attempted advanced statistical methods, such as machine learning, in large datasets and still failed to find robust predictors (Aderka, Kauffmann, Shalom, Beard, & Björgvinsson, 2021). One study, however, recently reported that pretreatment intraindividual variability was a significant predictor of sudden gains, with greater levels of symptom variability predicting sudden gains in an adult sample (Shalom et al., 2020) – although regular pretreatment measures may be more challenging to obtain, replication in a CYP sample is needed. Considering epilepsy‐specific factors, we assessed the impact that seizures had on CYP's life as a weekly session‐by‐session measure. However, we note that this is not synonymous with seizure frequency, and future studies may benefit from understanding the relationship between seizure frequency and sudden gains in therapy.

Experiencing a sudden gain in GBOs, parental self‐efficacy and SDQ S×S were associated with more favourable final‐session scores on the respective measures when compared to participants without sudden gains. However, sudden gains in any of the measures were not associated with the adjusted 6‐month SDQ total difficulties score (MICE trial primary outcome). Given that the RCT by Bennett et al. (2024) reported a clinically meaningful change in the SDQ measure of 5.3 points (*SD* 4.9) and that previous research indicates that the odds of psychiatric disorder decrease by 40% for each 2‐point decrease in the parent‐reported SDQ points (A. Goodman & Goodman, 2011), it is concluded that sudden gains are not required for a clinically meaningful change in the SDQ total difficulties score. This suggests that clinicians need not be disheartened if their patients make slow and steady progress (as opposed to sudden gains), as all positive change is beneficial (regardless of the rate of change).

These results show the importance of incorporating several constructs (including those more unique to LTCs) into session‐by‐session measurement to ensure important gains are not missed, although this must be balanced against questionnaire burden for participants. It must also be recognised that domain‐specific sudden gains may not impact standardised measures at later time points. Moreover, most studies, including this one, retrospectively analyse sudden gains upon therapy completion, when there is no further scope to improve outcomes. Future research should evaluate a focus on feedback of sudden changes to therapists and/or participants during therapy, with the potential to build on gains and improve outcomes further, augmenting the value of session‐by‐session measurement (Lutz, Schwartz, & Delgadillo, 2022).

This study has several strengths. It is the first to describe sudden gains in CYP receiving psychological therapy for mental health disorders in the context of an LTC (epilepsy). It is the largest in people with LTCs (of any age) and one of the largest in CYP more generally, including the widest paediatric age range, as well as those with autism spectrum disorder and intellectual disabilities – reflecting presentations seen in clinical practice. This study uniquely analysed multiple idiographic and standardised measures used as part of a modular intervention, whereas other studies typically used one or two measures only. Analyses were conducted blinded to MICE trial primary results by researchers independent of trial and therapy delivery, reducing bias.

This study also has limitations. Several measures had relatively narrow response scales and therefore RCIs were relatively small (often between 1 and 2 points). After rounding up to the nearest whole number, the minimum change defining ‘large absolute improvement’ (criterion one) was two points, potentially raising questions of clinical significance, an issue not unique to this study (Lutz et al., 2013). The magnitude of the sudden gains we identified were however noticeably larger than RCIs in all cases, with average magnitudes ranging from 2.74 to 4.06. For GBOs, other studies identified comparable RCIs of 2.45 (Edbrooke‐Childs et al., 2015) and 2.82 (O'Reilly et al., 2022). The parent‐reported SDQ total difficulties score was chosen as the mental health outcome as it was suitable for the full age range, intellectual ability range and range of clinical presentations within the sample. However, it is possible that specific subscales may have been more likely to be associated with sudden gains in certain session‐by‐session outcomes (e.g. the emotional subscale may have been associated with anxiety or depression session‐by‐session measures). We did not undertake such additional analyses due to the risk of Type I error. Similarly, whilst parent‐ and self‐report SDQ scores have been shown to correlate, a young person‐reported outcome may be differently associated with sudden gains (Hemmingsson, Ólafsdóttir, & Egilson, 2017).

We note the underrepresentation of participants residing in the lowest quintile of deprivation (9.4% vs. 26.8% from the upper quintile). We did not impute missing session‐by‐session data in order to avoid identifying potential sudden gains that were not reported by participants themselves, but using the average of the scores before and after a missing data point might have been even more conservative (Dour et al., 2013). A further potential limitation is that weekly measures were not collected in the trial control group. Collection of such data would have enabled assessment of whether therapy led to gains or losses over and above ‘normal’ variability without such intervention (or regression to the mean) – which could have been important given that sudden gains have been shown not to be unique to psychological interventions (Vittengl, Clark, & Jarrett, 2005). However, weekly outcome measurement is a substantial deviation from usual care which would have compromised the trial's scientific validity. Future research is needed to determine if and how sudden gains during psychological therapy are qualitatively different from those from other interventions (e.g. pharmacotherapy) and symptom variability without intervention.

## Conclusion

CYP with mental health disorders in the context of an LTC (epilepsy) frequently experienced sudden gains during a remotely delivered, evidence‐based, modular psychological intervention delivered by nonmental health professionals to CYP attending UK epilepsy services in an RCT. Across measures, 39% experienced between two and four sudden gains. We found some evidence for a small number of characteristics being associated with sudden gains in some measures. Sudden gains in certain measures were associated with better final‐session scores. Idiographic session‐by‐session measures with a focus on sudden gains may help to improve therapeutic outcomes. Future research and clinical practice should investigate how to increase the occurrence of sudden gains in CYP with long‐term conditions receiving psychological therapy.

## Author contributions

Conceptualisation: ARB, RS and SB. Data curation: ARB, RS, DL, MS and SB. Formal analysis: ARB and DL. Funding acquisition: ARB, RS, JHC, IH, ED, BC, SV and SB. Investigation: RS, IH and SB. Methodology: ARB, RS and SB. Project administration: ARB, RS and SB. Resources: RS and SB. Software: ARB and DL. Supervision: RS and SB. Validation: N/A. Visualisation: ARB. Writing – original draft: ARB, RS and SB. Writing – review and editing: ARB, RS, DL, JHC, IH, ED, BC, SV, MS and SB.

## Ethical considerations

MICE received ethical approval from South Central – Oxford A Research Ethics Committee (reference: 18/SC/0250). All participants, or their parents/legal guardians, provided written informed consent.

**Key points**

**What's known**

Sudden gains are common in psychological therapy and are associated with favourable outcomes, but no studies have investigated sudden gains in children and young people with mental health disorders in the context of a long‐term physical health condition.

**What's new**

We found that sudden gains occurred frequently across a range of measures during a remotely delivered, evidence‐based, modular psychological intervention delivered by nonmental health professionals to children and young people attending UK epilepsy services in an RCT. Sudden gains occurred most frequently when assessed using personalised measures and were associated with favourable outcomes at the end of therapy.

**What's relevant**

Personalised measures taken at each session with a focus on sudden gains may be a useful adjunct to treatment. Future research and clinical practice should investigate how to increase the occurrence of sudden gains in CYP with long‐term conditions receiving psychological therapy.

## Supporting information


**Appendix S1.** Supporting information.

## Data Availability

Data and code are not publicly available. Requests for access to the data from this study can be submitted via email to the corresponding authors with detailed proposals. Requests will be reviewed to verify whether they are subject to any intellectual property or confidentiality obligations. A signed data access agreement is required before accessing shared data.
