# Supplementary material for: Sudden gains in modular CBT for mental health disorders in children and young people with epilepsy
Source: J Child Psychol Psychiatry. 2025 Mar 20;66(10):1526–38. doi: 10.1111/jcpp.14164 (PMC12447670; doi:10.1111/jcpp.14164)
Supplement: Supplementary file 1 — Appendix S1. Supporting information. [file JCPP-66-1526-s001.docx]

# Supporting Information to

**“Sudden gains in modular CBT for mental health disorders
in children and young people with epilepsy.”**

Alvin Richards-Belle, Daniela Linton, J. Helen Cross, Isobel Heyman,
Emma Dalrymple, Bruce Chorpita, Sophia Varadkar, Mariam Shah,
MICE Study Group, Roz Shafran, Sophie Bennett

**Table of contents**

[Supplementary Introduction 2](#_Toc174198532)

[Table S1. Session-by-session measures in the MICE trial. 3](#_Toc174198533)

[Table S2. Number of participants with measures completed across clinical problem areas. 5](#_Toc174198534)

[Table S3. Sudden losses. 6](#_Toc174198535)

[Table S4. Number and percentage of participants with sudden gains across measures. 7](#_Toc174198536)

[Table S5. Number and percentage of participants with sudden losses across measures. 8](#_Toc174198537)

[Table S6. Adjusted associations between characteristics and occurrence of sudden gains across measures.* 9](#_Toc174198538)

[Supplemental References 11](#_Toc174198539)

Supplementary Introduction

*Critique of sudden gains operationalisation*

The concept of sudden gains was introduced by Tang and DeRubeis (1999), who proposed three criteria for identifying sudden gains - a change in symptom score must be large: 1) in absolute terms, 2) relative to previous symptom score, and 3) relative to symptom fluctuation. In their inceptual study of 61 adults receiving CBT for depression, the criteria were operationalised as: 1) an improvement of ≥7 points on the Beck Depression Inventory (BDI), 2) at least 25% of the pre-gain BDI score, and 3) the mean of the BDI scores from the three sessions prior to the gain must be significantly greater than the mean from the three after, determined using a *t*-test (*p*<0.05) (Tang & DeRubeis, 1999).

Subsequent authors have adapted, criticised, and revised the original criteria/operationalisation of sudden gains as proposed by Tang and DeRubeis (1999). For example, to study measures other than the BDI, the reliable change index (RCI) (Jacobson & Truax, 1991) is typically used as the critical value defining large absolute improvement (criterion one) (Stiles et al., 2003). Some have argued that a change of 25% from the pre-gain score (criterion two) incorrectly assumes that symptom scales are ratio measures and that this criterion contributes little overall discriminatory value (Hardy et al., 2005). Although criterion three is considered most stringent (Dour et al., 2013; Stiles et al., 2003), it has been criticised on the basis that the three pre- and post-gain scores are not independent observations and therefore a t-test is not statistically appropriate; authors have used alternative *p*- or *t*-statistic values as cut-points (including to account for missing data) (Hardy et al., 2005; Lutz et al., 2013; Wiedemann et al., 2020).

Table S1. Session-by-session measures in the MICE trial.

| **Focus area** | **Scale name** | **Items** | **Frequency** | **Scale** | **Per session scoring** | **Calculated reliable change index** | **Notes** |
| --- | --- | --- | --- | --- | --- | --- | --- |
| Idiographic goals | Goal-based outcomes (GBOs) | 1  (per goal) | Every session | 1=low progress, 10=high progress. | N/A | 0.82^1^ | Rate progress towards up to three goals.^2^ |
| Disruptive behaviour | Oppositional Defiant Disorder – Parent report (Child Outcomes Research Consortium, 2023) | 8 | As relevant | 0=not true, 1=somewhat true, 2=certainly true. | Sum to overall score. | 1.52 | Statement about behaviour/conduct problems over the last week. Items based on Oppositional Defiant Disorder DSM-IV criteria. |
| Generalised anxiety | GAD subscale (RCADS) (Chorpita et al., 2000) | 6 | As relevant | 0=never, 1=sometimes, 2=often, 3=always. | Sum to overall subscale score. | 1.97 | Anxiety items, over the last week. |
| Separation anxiety | Separation anxiety disorder subscale (RCADS) (Chorpita et al., 2000) | 7 | As relevant | 0=never, 1=sometimes, 2=often, 3=always. | Sum to overall subscale score. | 1.92 | Separation anxiety items, over the last week. |
| Panic | Panic disorder subscale (RCADS) (Chorpita et al., 2000) | 9 | As relevant | 0=never, 1=sometimes, 2=often, 3=always. | Sum to overall subscale score. | --^3^ | Panic items, over the last week. |
| Social anxiety | Social phobia subscale (RCADS) (Chorpita et al., 2000) | 9 | As relevant | 0=never, 1=sometimes, 2=often, 3=always. | Sum to overall subscale score. | -^3^ | Social anxiety items, over last week. |
| Depression | MDD subscale (RCADS) (Chorpita et al., 2000) | 10 | As relevant | 0=never, 1=sometimes, 2=often, 3=always. | Sum to overall subscale score. | --^3^ | Depression items, over the last week. |
| Seizure impact | Seizure impact | 1 | Every session | 1=no impact, 10=significant negative impact. | N/A | 1.60 | “How much of an impact have my child’s seizures had on my child’s life?” |
| Parental self-efficacy | Brief Parental Self Efficacy Scale (BPSES) (Woolgar et al., 2023) | 5 | Every session^4^ | 1=strongly disagree, 2=disagree, 3=neutral, 4=agree, 5=strongly agree. | Sum to overall score. | 1.43 | 5 statements, focused on behaviour management. |
| SDQ SxS | SDQ SxS scale (Goodman, 1997) | 5 | Every session | 0=not at all, 1=only a little, 2=a medium amount, 3=a great deal. | Sum to overall score. | 1.90 | The original SDQ SxS scale uses the following response scale: 0=not at all, 0=only a little, 1=a medium amount, 2=a great deal. We used a modified response scale in order to detect changes across the lowest two levels. |

*GBO, goal-based outcome; RCADS, Revised Child Anxiety and Depression Scale; SDQ, strengths and difficulties questionnaire.*

^1^ Although a reliable change index of <1 was identified for GBO mean, we used an RCI of 2 as a change of a less than or equal to 1 was not felt to be clinically significant.

^2^ a fourth goal was developed and rated for 3 participants; these scores were not included in this study.

^3^ the reliable change index was not calculated due to insufficient participant numbers.

^4^ not completed if sessions were carried out with the child/young person directly.

Table S2. Number of participants with measures completed across clinical problem areas.

| **Problem areas*** | **N** | **%** |
| --- | --- | --- |
| Disruptive behaviour | 105 | 71.4% |
| Generalised anxiety | 59 | 40.1% |
| Separation anxiety | 27 | 18.4% |
| Depression | 19 | 12.9% |
| Social anxiety | 5 | 3.4% |
| Panic | 2 | 1.4% |

* Problem areas are not mutually exclusive. Participants completed measures if and when relevant to the participant’s therapy.

Table S3. Sudden losses.

| **Domain** | **N^1^** | **Session Intervals analysed, n** | **Total, n** | **Occurrence rate, n (%)** | **Session no, mode** | **Multiple,  n (%)** | **Magnitude, mean (SD)** | **Reversal,  n (%)** |
| --- | --- | --- | --- | --- | --- | --- | --- | --- |
| GBO mean | 147 | 1898 | 7 | 7 (4.76) | 8 | 0 (0.00) | -3.26 (1.74) | 6 (85.71) |
| Disruptive behaviour | 105 | 1065 | 19 | 18 (17.14) | 13 | 1 (0.95) | -3.67 (2.03) | 9 (50.00) |
| Generalised anxiety | 59 | 605 | 11 | 10 (16.95) | 2 | 1 (1.69) | -3.80 (1.55) | 8 (80.00) |
| Separation anxiety | 27 | 247 | 2 | 2 (7.41) | 10 | 0 (0.00) | -6.50 (6.36) | 1 (50.00) |
| Seizure impact | 140 | 1427 | 26 | 22 (15.71) | 12 | 4 (2.86) | -3.45 (1.57) | 17 (77.27) |
| Parental self-efficacy | 120 | 1258 | 0 | 0 (0) | - | - | - | - |
| SDQ SxS | 137 | 1292 | 18 | 17 (12.41) | 13 | 1 (0.73) | -3.88 (2.26) | 11 (64.71) |

*GBO, goal-based outcome; SDQ, strengths and difficulties questionnaire.*

^1^ Participants are included in the n for a specific measure if they received at least 6 sessions, had not withdrawn consent and had >1 response on the relevant scale through the course of therapy.

Table S4. Number and percentage of participants with sudden gains across measures.

| **Number of  sudden gains across measures*** | **N** | **%** |
| --- | --- | --- |
| 0 | 29 | 19.7% |
| 1 | 58 | 39.5% |
| 2 | 32 | 21.8% |
| 3 | 16 | 10.9% |
| 4 | 9 | 6.1% |
| 5 | 2 | 1.4% |
| 6 | 1 | 0.7% |
| 7 | 0 | 0.0% |
| Sum | 147 | 100% |

* Considers GBO mean, disruptive behaviour, generalised anxiety, separation anxiety, parental self-efficacy, seizure impact and SDQ SxS. The maximum possible would be 7.

Table S5. Number and percentage of participants with sudden losses across measures.

| **Number of  sudden losses across measures*** | **N** | **%** |
| --- | --- | --- |
| 0 | 89 | 60.5% |
| 1 | 45 | 30.6% |
| 2 | 9 | 6.1% |
| 3 | 3 | 2.0% |
| 4 | 1 | 0.7% |
| 5 | 0 | 0.0% |
| 6 | 0 | 0.0% |
| 7 | 0 | 0.0% |
| Sum | 147 | 100% |

* Considers GBO mean, disruptive behaviour, generalised anxiety, separation anxiety, parental self-efficacy, seizure impact and SDQ SxS. The maximum possible would be 7.

Table S6. Adjusted associations between characteristics and occurrence of sudden gains across measures.*

| **Predictor/Domain** | **GBO mean** | **Disruptive behaviour** | **Generalised anxiety** | **Parental self-efficacy** | **Seizure impact** | **SDQ SxS** |
| --- | --- | --- | --- | --- | --- | --- |
| Sex: Male (vs Female) | 0.71  (0.33, 1.50) [0.37] | 0.88  (0.35, 2.21) [0.79] | 1.18  (0.22, 6.48) [0.85] | 1.45  (0.60, 3.55) [0.41] | 1.44  (0.54, 3.96) [0.47] | 1.00  (0.44, 2.31) [>0.99] |
| Age: >=11y (vs <11y) | 1.23  (0.57, 2.66) [0.59] | 0.83  (0.30, 2.17) [0.71] | 2.14  (0.38, 15.5) [0.41] | 0.42  (0.16, 1.03) [0.064] | 0.77  (0.28, 2.03) [0.59] | 1.12  (0.48, 2.61) [0.80] |
| Ethnicity: Other (vs White)^1^ | 0.27  (0.10, 0.67) [0.007] | 0.55  (0.17, 1.62) [0.29] | 0.52  (0.05, 3.79) [0.54] | 1.82  (0.71, 4.71) [0.21] | 1.44  (0.43, 4.47) [0.53] | 0.55  (0.18, 1.47) [0.25] |
| ASD: present (vs absent) | 1.46  (0.59, 3.68) [0.42] | 1.51  (0.54, 4.18) [0.43] | 0.68  (0.06, 6.55) [0.75] | 1.49  (0.56, 3.96) [0.42] | 1.46  (0.45, 4.50) [0.51] | 0.93  (0.34, 2.42) [0.88] |
| ID: present (vs absent) | 0.68  (0.30, 1.48) [0.33] | 2.62  (1.03, 7.01) [0.046] | 0.43  (0.06, 2.56) [0.37] | 1.34  (0.56, 3.27) [0.51] | 0.45  (0.15, 1.22) [0.13] | 1.19  (0.50, 2.84) [0.69] |
| Primary mental health disorder: Disruptive behaviour (vs other) | 1.31  (0.60, 2.89) [0.50] | 0.77  (0.26, 2.36) [0.64] | 5.07  (0.87, 34.7) [0.077] | 0.89  (0.36, 2.23) [0.81] | 1.14  (0.41, 3.23) [0.81] | 0.84  (0.35, 2.03) [0.70] |
| Total number of sessions | 1.04  (0.95, 1.15) [0.38] | 1.09  (0.98, 1.23) [0.14] | 1.40  (1.11, 1.86) [0.010] | 1.18  (1.06, 1.34) [0.004] | 1.16  (1.01, 1.35) [0.041] | 1.07  (0.96, 1.20) [0.23] |
| Caregiver employment status:  Not employed (vs employed)^2^ | 1.46  (0.70, 3.06) [0.32] | 1.11  (0.44, 2.76) [0.82] | 0.11  (0.01, 0.70) [0.039] | 0.79  (0.32, 1.87) [0.59] | 1.34  (0.50, 3.53) [0.55] | 1.25  (0.55, 2.83) [0.59] |
| Pre-treatment score | 0.83  (0.63, 1.09) [0.20] | 1.07  (0.95, 1.21) [0.25] | 1.12  (0.90, 1.43) [0.31] | 0.97  (0.86, 1.10) [0.66] | 1.32  (1.13, 1.57) [<0.001] | 0.95  (0.85, 1.06) [0.34] |

*GBO, goal-based outcome; SDQ, strengths and difficulties questionnaire.*

^*^ Estimates are Adjusted Odds Ratio (95% CI) [p value]. All models are adjusted for: sex, age group, ethnicity, presence of autism spectrum disorder, presence of intellectual disability, total number of sessions, primary mental health disorder (disruptive behaviour vs anxiety/depression), employment status of primary caregiver, and the relevant pre-treatment score.

^1^ participants who did not disclose this information were included in the ‘other’ category.

^2^ full-time, part-time, and self-employed caregivers were all considered as employed.

Supplemental References

Child Outcomes Research Consortium. (2023, December). *How are things? (Behavioural difficulties, Oppositional Defiant Disorder - ODD-p)*. https://www.corc.uk.net/outcome-experience-measures/how-are-things-behavioural-difficulties-oppositional-defiant-disorder-odd-p/

Chorpita, B. F., Yim, L., Moffitt, C., Umemoto, L. A., & Francis, S. E. (2000). Assessment of symptoms of DSM-IV anxiety and depression in children: A revised child anxiety and depression scale. *Behaviour Research and Therapy*, *38*(8), 835–855. https://doi.org/10.1016/s0005-7967(99)00130-8

Dour, H. J., Chorpita, B. F., Lee, S., & Weisz, J. R. (2013). Sudden gains as a long-term predictor of treatment improvement among children in community mental health organizations. *Behaviour Research and Therapy*, *51*(9), 564–572. https://doi.org/10.1016/j.brat.2013.05.012

Goodman, R. (1997). The Strengths and Difficulties Questionnaire: A research note. *Journal of Child Psychology and Psychiatry, and Allied Disciplines*, *38*(5), 581–586. https://doi.org/10.1111/j.1469-7610.1997.tb01545.x

Hardy, G. E., Cahill, J., Stiles, W. B., Ispan, C., Macaskill, N., & Barkham, M. (2005). Sudden gains in cognitive therapy for depression: A replication and extension. *Journal of Consulting and Clinical Psychology*, *73*(1), 59–67. https://doi.org/10.1037/0022-006X.73.1.59

Jacobson, N. S., & Truax, P. (1991). Clinical significance: A statistical approach to defining meaningful change in psychotherapy research. *Journal of Consulting and Clinical Psychology*, *59*(1), 12–19. https://doi.org/10.1037//0022-006x.59.1.12

Lutz, W., Ehrlich, T., Rubel, J., Hallwachs, N., Röttger, M.-A., Jorasz, C., Mocanu, S., Vocks, S., Schulte, D., & Tschitsaz-Stucki, A. (2013). The ups and downs of psychotherapy: Sudden gains and sudden losses identified with session reports. *Psychotherapy Research*, *23*(1), 14–24. https://doi.org/10.1080/10503307.2012.693837

Stiles, W. B., Leach, C., Barkham, M., Lucock, M., Iveson, S., Shapiro, D. A., Iveson, M., & Hardy, G. E. (2003). Early sudden gains in psychotherapy under routine clinic conditions: Practice-based evidence. *Journal of Consulting and Clinical Psychology*, *71*(1), 14–21.

Tang, T. Z., & DeRubeis, R. J. (1999). Sudden gains and critical sessions in cognitive-behavioral therapy for depression. *Journal of Consulting & Clinical Psychology*, *67*(6), 894–904.

Wiedemann, M., Thew, G. R., Stott, R., & Ehlers, A. (2020). suddengains: An R package to identify sudden gains in longitudinal data. *PLOS ONE*, *15*(3), e0230276. https://doi.org/10.1371/journal.pone.0230276

Woolgar, M., Humayun, S., Scott, S., & Dadds, M. R. (2023). I Know What to Do; I Can Do It; It Will Work: The Brief Parental Self Efficacy Scale (BPSES) for Parenting Interventions. *Child Psychiatry & Human Development*. https://doi.org/10.1007/s10578-023-01583-0
